# Conformational and Immunogenicity Studies of the *Shigella flexneri* Serogroup 6 O-Antigen: The Effect of O-Acetylation

**DOI:** 10.3390/vaccines9050432

**Published:** 2021-04-27

**Authors:** Nicole Inge Richardson, Neil Ravenscroft, Vanessa Arato, Davide Oldrini, Francesca Micoli, Michelle M. Kuttel

**Affiliations:** 1 Department of Chemistry, University of Cape Town, Rondebosch 7701, South Africa; nicole.richardson.za@gmail.com (N.I.R.); neil.ravenscroft@uct.ac.za (N.R.); 2 GSK Vaccines Institute for Global Health (GVGH) S.r.l., via Florentina 1, 53100 Siena, Italy; vanessa.x.arato@gsk.com (V.A.); davide.x.oldrini@gsk.com (D.O.); francesca.x.micoli@gsk.com (F.M.); 3 Department of Computer Science, University of Cape Town, Rondebosch 7701, South Africa

**Keywords:** O-antigen, conformation, *Shigella flexneri*, molecular modeling, GMMA, O-acetylation

## Abstract

The pathogenic bacterium *Shigella* is a leading cause of diarrheal disease and mortality, disproportionately affecting young children in low-income countries. The increasing prevalence of antibiotic resistance in *Shigella* necessitates an effective vaccine, for which the bacterial lipopolysaccharide O-antigen is the primary target. *S. flexneri* serotype 6 has been proposed as a multivalent vaccine component to ensure broad protection against *Shigella*. We have previously explored the conformations of *S. flexneri* O-antigens from serogroups Y, 2, 3, and 5 that share a common saccharide backbone (serotype Y). Here we consider serogroup 6, which is of particular interest because of an altered backbone repeat unit with non-stoichiometric O-acetylation, the antigenic and immunogenic importance of which have yet to be established. Our simulations show significant conformational changes in serogroup 6 relative to the serotype Y backbone. We further find that O-acetylation has little effect on conformation and hence may not be essential for the antigenicity of serotype 6. This is corroborated by an in vivo study in mice, using Generalized Modules for Membrane Antigens (GMMA) as O-antigen delivery systems, that shows that O-acetylation does not have an impact on the immune response elicited by the *S. flexneri* serotype 6 O-antigen.

## 1. Introduction

Diarrheal disease is the eighth most common cause of death world-wide, with the highest mortality in infants and geriatrics [1,2,3]. Diarrheal disease disproportionately affects low-income regions: approximately 90% occurs in south Asia and sub-Saharan Africa [2,3]. After rotavirus, the pathogenic Gram-negative bacterium *Shigella* is the primary cause of diarrheal mortality, so the increasing prevalence and antibiotic resistance of *Shigella* is a cause for concern [2,3,4,5,6,7,8]. As a vaccine has been licensed for rotavirus, *Shigella* is a focus of current vaccine development [2,6].

The O-antigen (O-Ag) on the bacterial cell surface lipopolysaccharide is the primary target of the host immune response against *Shigella* and the focus of current vaccine design [4,6,9]. On the basis of O-Ag composition, the *Shigella* species is divided into the four subgroups *Shigella dysenteriae*, *Shigella flexneri*, *Shigella sonnei*, and *Shigella boydii* [6,10] *S. flexneri* is the most common subgroup to cause disease (66%), followed by *S. sonnei* (24%), *S. dysenteriae* (5%), and *S. boydii* (5%) [9]. For *S. flexneri*, five serotypes—2a, 6, 3a, 2b, and 1b—account for almost 90% of disease [6,9]. Based on analysis of structural similarities, it has been proposed that a tetravalent vaccine containing *S. flexneri* serotypes 2a, 3a, 6, and *S. sonnei*, would provide direct protection against 75% of *S. flexneri* disease, with potential for broader coverage against non-vaccine serotypes (from shared group antigens) as high as 90% [4,6,9].

**Figure 1:**
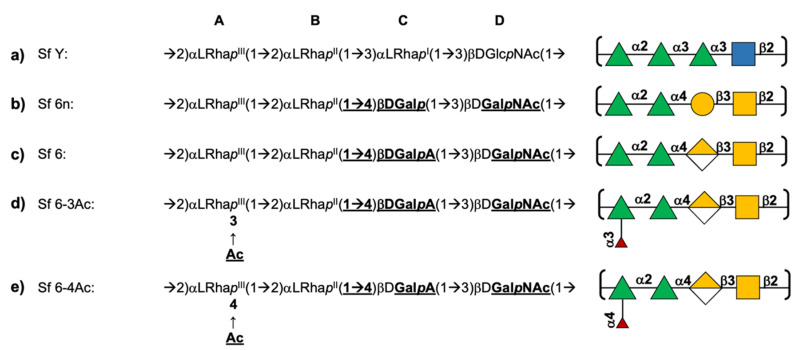
Primary line structures (differences between serotypes shown in bold) and schematic diagrams of the O-antigen repeating units of *S. flexneri* modelled in this work: (**a**) serotype Y (Sf Y), (**b**) non-biological serotype 6n (Sf 6n) with Gal instead of GalA, (**c**) serotype 6 (Sf 6), (**d**) serotype 6 with 100% O-acetylation at O3 of Rha^III^ (Sf 6-3Ac), and (**e**) serotype 6 with 100% O-acetylation at O4 of Rha^III^ (Sf 6-4Ac). Diagrams use the Symbol Nomenclature for Glycans (SNFG) [12,13] with green triangle for Rha, blue square for GlcNAc, blue circle for Glc, yellow circle for Gal, yellow half diamond for GalA, yellow square for GalNAc, and red triangle for O-acetyl.

*S. flexneri* is differentiated into serogroups based on type O-factor groups (I; II; III; IV; V; VI; VII) and further into serotypes based on group O-factors (3,4; 6; 7, 8; 9; 10; IV-1) that are determined by phage-mediated glucosylation, phosphorylation, and O-acetylation of the O-Ag [10,11]. Apart from serogroup 6, all of the *S. flexneri* serotypes share a common backbone repeating unit (serotype Y (Sf Y), Figure 1a) [10,11] comprising four residues: three rhamnoses (Rha) and a single N-acetylglucosamine (GlcNAc). The serogroup 6 (Sf 6) backbone repeating unit consists of two Rha residues, a galacturonic acid (GalA), and an N-acetylgalactosamine (GalNAc) residue (Figure 1c) [10,11] and is acidic due to the presence of the charged GalA residue.

Sf 6 has only one structural modification characterized: a phage-mediated, random, and non-stoichiometric O3/O4 acetylation of Rha^III^ (residue A, Figure 1d,e) [10,14]. Modification on O3 confers group O-factor 9 onto Sf 6 and is also observed in serotypes 1b, 2a, 5a, 7a, Y1, and Y2 [10,15]. An important question in the context of the O-Ag as a vaccine target is whether O-acetylation is important for antigenicity and immunogenicity; in the case of *S. flexneri* serotype 2a (Sf 2a), O-factor 9 has been shown to not add to broad antibody recognition [16].

When it is present, cross-protection between antigens allows for reduction of the number of costly vaccine components [17]. Cross-reactivity between serotypes is expected when a shared epitope is present and, although Sf 6 has a different backbone to the non-serotype 6 backbones, cross reactivity between Sf 2a and Sf 6 is expected, due to the shared O-factor 9 epitope. In clinical trials, vaccination with an O-acetylated Sf 2a conjugate in humans (children and adults) induced protective antibodies against Sf 6 [18]. The reverse was also seen in two cases where children (unvaccinated with Sf 2a) who developed Sf 6 disease subsequently developed protective antibodies against Sf 2a [18]. However, data from mouse models showed no cross-reactivity between Sf 6 and Sf 2a [18].

Molecular modeling of antigen conformations can provide insight into the likelihood of cross-protection between *S. flexneri* O-Ags [15,19]. We have previously modelled the conformations of *S. flexneri* serogroup Y, 2, 3, and 5 O-antigens [15,19]. The molecular dynamics simulations revealed that *S. flexneri* serotypes are highly flexible, with a wide distribution of conformations. Substitutions of the backbone residues (glucosylation and/or O-acetylation) were found to limit the flexibility and distribution of conformations to varying degrees and we proposed three guiding heuristics to describe and predict the effect of substitutions [19]. Particularly of relevance to Sf 6 is that we found that substitution with O-factor 9 restricts the O-Ags to predominantly helical conformations. Conformational differences highlighted by this work support the inclusion of both serotypes 2a and 3a in a potential vaccine. We now focus on Sf 6 (type O-factor VI; group O-factor 9), the second most prevalent cause of *S. flexneri* disease in low- and middle-income countries [9].

In this work we performed molecular dynamics (MD) simulations of the five structures listed in Figure 1: the common Sf Y backbone (Figure 1a); a neutral (non-biological) serotype 6n in which GalA is replaced with Gal (Sf 6n; Figure 1b); Sf 6 (Figure 1c); as well as Sf 6 with 100% O-acetylation at either O3 of Rha^III^ (Sf 6-3Ac, Figure 1d) or O4 of Rha^III^ (Sf 6-4Ac, Figure 1e). The specific aims of this study are to compare: (a) the conformation and dynamics of the unique Sf 6 backbone with the common Sf Y backbone; (b) the conformation of Sf 6 with the neutral Sf 6n, to establish the effect of charge on conformation; and (c) the effect of O-acetylation at O3 or O4 of Rha^III^ (residue A) on Sf 6 conformation.

To corroborate our modeling results on O-acetylation, we performed immunological studies in mice with the aim of understanding whether the presence of the O-acetyl group has an impact on *S. flexneri* 6 O-Ag immunogenicity. We genetically manipulated wild type Sf 6 by deleting the *oacC* gene which codes for the O-acetyltransferase C enzyme that is responsible for the O-acetylation of the O-Ag on Rha^III^ (residue A). Mice were then immunized using Sf 6 Generalized Modules for Membrane Antigens (GMMA) [20] (a proposed alternative delivery system for *Shigella* O-Ags [21,22]) obtained from either the wild-type or the *oacC* knock-out *S. flexneri* 6 strains [23] and the immune responses compared.

## 2. Materials and Methods

### 2.1. Molecular Simulations

We used our established systematic approach to the modeling of polysaccharides, as follows [19,24]. To identify the preferred orientation of each glycosidic linkage, two-dimensional φ, ψ potential mean force (PMF) calculations were performed for the glycosidic linkages of all the disaccharide fragments of the O-Ag repeating units. The glycosidic linkage orientations are generally defined by two dihedral angles, φ = H1-C1-O1-Cx’ and ψ = C1-O1-Cx’-Hx’, which are equivalent φ and ψ in IUPAC nomenclature [15].

The preferred glycosidic linkage orientations identified by the PMFs were then used to build three repeating unit (3 RU) O-Ag chains for initial Molecular Dynamics (MD) simulations in aqueous solution (data not shown), which were then extended to six repeating unit (6 RU) chains. Chain length is an important consideration when modeling O-Ags, as a short chain may have insufficient molecular flexibility while long chains become extremely time consuming and too computationally expensive to model. *S. flexneri* exhibits a range of O-Ag chain lengths, however, 3RU is considered sufficient to represent O-Ag conformation (based on our previous work with *S. flexneri* and studies in mice) [15,19,20,25,26]. Furthermore, antibodies have been shown to only bind small fragments of the O-Ag between one and seven residues in length corresponding to 1–2 RU in the case of *S. flexneri* [27].

Following initial system equilibration, molecular dynamics runs of 1–2 µs were performed and data analyses were performed on these production runs.

#### 2.1.1. PMF Calculations

Disaccharide structures were built using CarbBuilder and visualized with Visual Molecular Dynamics (VMD) software [28,29,30]. PMF calculations were performed using the Metadynamics package incorporated into the Nanoscale Molecular Dynamics (NAMD) software [31]. The φ and ψ dihedrals used as collective variables to establish preferred conformations for rotation about the φ and ψ dihedrals of the glycosidic linkages in the disaccharide units.

For all neutral disaccharides, calculations were performed in vacuum (gas phase). For all charged disaccharides, calculations were performed in solution, using the TIP3P [32] model of water with a sodium counter-ion to ensure system neutrality (a requirement for the calculations of electrostatic interactions to converge). Systems were solvated and ionized using VMD’s built in solvation and ionization tools.

Each disaccharide system was run at 300 K for 1000 ns (vacuum simulations) or until the biasing energy reached at least 10 kcal.mol^−1^ (solution simulations). Once the runs were complete, internal scripts were used to extract global and local minimum energies and to generate contour plots of φ versus ψ.

#### 2.1.2. Molecular Dynamics

Simulations were run using NAMD (version 2.13) with CUDA extensions for utilizing graphics processors for parallel computing [33]. The CHARMM36 additive force field was chosen for the simulations [34,35].

The data for Sf Y from our previous work [15] was extended by 1000 ns and included in this study. Starting structures of the Sf 6 O-Ags (Figure 1) were built with CarbBuilder using the global minima for the glycosidic linkages determined from the PMF calculations. The starting structures were subsequently minimized using NAMD for 10,000 steps at 310 K.

Minimized structures were solvated in cubic TIP3P [32] water boxes of 90 Å per side. The charged Sf 6, Sf 6-3Ac and Sf 6-4Ac systems underwent an ionization step to add six sodium (Na^+^) counter ions to neutralize the system. Initial minimization and heating protocols comprised 5 K incremental temperature reassignments beginning at 10 K up to 310 K with 5000 steps of NAMD minimization and 2000 steps of MD at each temperature reassignment.

Periodic boundary conditions equivalent to the cubic box size were employed for the solvated simulation with wrapping on. Long range electrostatics were implemented with Particle Mesh Ewald summation (PME) on a 90 Å grid size [36]. Of the pairs, 1–3 were excluded from non-bonded interactions, 1–4 interactions were scaled by a factor of 1, and a dielectric constant of 1 was used for the system. Smoothing functions were applied to both the electrostatics and van der Waals forces with switching and cut-off distances of 15 Å and 12 Å respectively.

A Leap-Frog Verlet integrator was used to integrate the equations of motion over a step size of 1 fs. A distance of 18 Å was used as the cut-off for inclusion in the pair list for calculation of non-bonded forces. The short-range non-bonded interactions were calculated every 1 fs, full electrostatics calculations were performed every 2 fs, and atoms were reassigned every 10 fs [37].

Simulations were sampled under isothermal-isobaric (nPT) ensemble. Langevin dynamics were used to control the temperature with a damping coefficient of 5/ps. Nosé-Hoover Langevin piston dynamics were used as a barostat to maintain a target pressure of 1 atm. Variable system volume was used with a piston period of 100 fs and decay of 50 fs. Post equilibration (200 ns), simulations underwent production runs of 1800 ns for Sf Y and Sf 6, 800 ns for Sf 6n, and 900 ns Sf 6-3Ac and Sf 6-4Ac as different simulation lengths were required for the different models to appear converged.

#### 2.1.3. Simulation Convergence

We addressed the convergence using the method of block standard averaging, applied to two measurables: molecular end-to-end distance and radius of gyration (see Supplementary Figure S1), as previously described, and implemented the method using in-house Python scripts [19,38].

For all serotypes modeled, the blocked standard error (BSE) can be seen to have reached plateaus. The simulation lengths were large multiples of the correlation times (Sf Y, 50 ns; Sf 6n, 24 ns; Sf 6, 6 ns; Sf 6-3Ac, 11 ns; Sf 6-4Ac,18 ns) and numbers of independent samples (Sf Y, 40; Sf 6n, 83; Sf 6, 180; Sf 6-3Ac, 102; Sf 6-4Ac, 60) were >>1. Furthermore, the equilibration time of 200 ns was >> the correlation time. Thus, the simulations appear converged and 200 ns equilibration is sufficient. For Sf Y and Sf 6, longer simulation times were required for convergence (both 2000 ns) than for Sf 6n (1000 ns), Sf 6-3Ac (1100 ns), and Sf 6-4Ac (1100 ns).

#### 2.1.4. Data Analysis

Output trajectories were extracted every 25 ps and analyzed at 250 ps intervals. Inter-atomic distances and dihedral angles were measured using VMD’s Tk console and graphical user interface (GUI) with data analyses performed using in-house Python scripts and plots generated using Matplotlib [39].

The end-to-end distance, *r*, was measured from C2 of Rha^II^ (residue B) at the non-reducing end, to C1 of Rha^I^/GalA (residue C) at the reducing end, thus excluding the highly flexible terminal residues.

The dihedral angles for each glycosidic linkage were measured as a combination of the dihedrals from the central repeating units, 3 and 4, thus providing a sample of the most central angles.

Molecular conformations were visualized using VMD and clustering of production run trajectories were performed using the WMC PhysBio GUI for VMD’s built-in cluster command [40]. Prior to clustering, the molecules were aligned on the ring and linkage atoms of the least flexible central repeating unit, RU 3. Clustering was performed on the ring and linkage atoms in RUs 2, 3, 4, and 5, avoiding the highly flexible terminal repeating units 1 and 6. A cut-off of 5.5 Å was set and clusters < 6% were discarded.

Hydrophilic/hydrophobic regions of the molecular surface were analyzed using VMD’s built in “measure sasa” command. The solvent accessible surface area (sasa) analysis was performed by probing first hydrophilic regions (comprising hydroxyl groups, carbonyl groups, amine groups, ring oxygens, and linkage oxygens) and then hydrophobic/neutral regions (comprising methyl groups, CH_2_ groups, ring carbons, and ring protons) of the molecule using a van der Waal’s radius of 1.4 Å-analogous to that of water. The ratio of hydrophilic to hydrophobic/neutral regions was then calculated to determine the percentage hydrophilic surface area available for potential antibody binding.

When necessary, carbohydrate rings were visualized using the PaperChain visualization algorithm and the hydrophilic and hydrophobic surfaces were visualized using the Quicksurf visualization algorithm [41,42].

### 2.2. Immunological Studies

We compared the immune responses in vaccinated mice and their functional activity against both Sf 6 and de-O-acetylated Sf 6 strains. We genetically manipulated wild type Sf 6 by deleting the *oacC* gene which codes for the O-acetyltransferase C enzyme that is responsible for the O-acetylation of the O-Ag on Rha^III^ (residue A). Mice were then immunized using Sf 6 GMMA [20] obtained from either the wild-type or the *oacC* knock-out *S. flexneri* 6 strains [23]. GMMA are Outer Membrane Vesicles (OMV) naturally released from Gram-negative bacteria mutated to increase OMV yield, proposed as an alternative delivery system for *Shigella* O-Ags [21,22].

#### 2.2.1. Bacterial Strains, Mutant Generation and Growth Conditions

A *S. flexneri* 6 wild type strain was obtained from the Wellcome Trust Sanger Institute and Public Health England [43]. Strain Sf6_Sh10.8537 was selected for the generation of deletion mutants. To generate the mutants, the kanamycin resistance gene *aph* was used to replace the *tolR* and the *oacC* genes. The resistance cassette replacement constructs were amplified from the pKD4 vector using the following primers: Fw ATGTTTGAAATTGATAGCCTATTATTAATAACATCCGTGATAATCTTGTCGTGTAGGCTGGAGCTGCTTC and Rv GGTTTGTTTTGTTATATTAATGAAAGGTAGTTCAATTAATTTAAATGTTACATATGAATATCCTCCTTAG. PCR products were used to transform recombination-prone *S. flexneri* 6 recipient cells carrying pKD46 as described previously [44]. The *oacC* gene was also deleted in a non-Δ*tolR* background (not overblebbling) for use as a target strain in SBA experiments.

All bacterial strains were grown at 30 °C in liquid Luria–Bertani (LB) medium in a rotary shaker for 16 h. For GMMA production, overnight cultures were diluted in HTMC medium (15 g/L glycerol, 30 g/L yeast extract, 0.5 g/L MgSO4, 5 g/L KH_2_PO_4_, and 20 g/L K_2_HPO_4_) to an optical density at 600 nm (OD600) of 0.3 and grown at 30 °C in a rotary shaker for 8 h using baffled flasks with a liquid to air volume ratio of 1:5.

#### 2.2.2. GMMA Production and Characterization

After growth, bacteria were pelleted through centrifugation at 5000× *g* for 45 min at 4 °C. Cell-free supernatants were ultracentrifuged at 175,000× *g* for 2 h at 4 °C; the resulting pellet, containing GMMA, was washed with phosphate-buffered saline (PBS), further ultra-centrifuged at 175,000× *g* for 2 h at 4 °C and finally resuspended in PBS.

GMMA purity was assessed by HPLC–SEC analysis [45]; total protein content was estimated by micro BCA (Thermo Scientific, Waltham, MA, USA); O-Ag sugar content was quantified by determination of methyl pentoses (6-deoxyhexoses) with the Dische colorimetric method [46].

O-Ag extraction and purification from GMMA was performed as previously described [45].

Nuclear magnetic resonance (NMR) spectroscopy was used to confirm O-Ag identity and to calculate the degree of O-acetylation [11]. All NMR spectra were acquired at 50 °C with an AEON AVANCE III 600 MHz spectrometer (Bruker, Billerica, MA, USA) equipped with a high-precision temperature controller using a 5 mm QCI CryoProbe as previously described [20].

#### 2.2.3. Immunogenicity Studies in Mice

Animal studies were performed at the GSK Animal Care Facility under the animal project 526/2020-PR 26/05/2020, approved by the Italian Ministry of Health. All animal studies were ethically reviewed and carried out in accordance with European Directive 2010/63/EEC and the GSK policy on the Care, Welfare and Treatment of Animals. Five-week-old female wild-type mice were immunized intraperitoneally with 200 μL of vaccine at days 0 and 28. Sera were collected at day 42. Different O-Ag doses were tested in the range 0.005–0.5 μg.

Individual mouse sera were tested for anti-O-Ag total IgG by enzyme-linked immunosorbent assay (ELISA), as previously described [47]. *S. flexneri* 6 Group 4 Capsule (G4C) [20], sharing the same O-Ag RU, at a concentration of 5 μg/mL in carbonate buffer pH 9.6, was used as a coating antigen. Single sera were tested against both a wild-type and an *oacC* knock-out *S. flexneri* 6 strain in a serum bactericidal assay (SBA) based on luminescent readout as described previously [20,48].

Results of the assay were expressed as the IC50: the reciprocal serum dilution that produced a 50% reduction of luminescence, which corresponds to 50% growth inhibition of the bacteria present in the assay [49,50]. GraphPad Prism 7 software (GraphPad Softare, San Diego, California) was used for curve fitting and IC50 determination. Titers below the minimum measurable signal were assigned a titer of 50, corresponding to half of the first dilution of sera tested.

Statistical analysis was performed using GraphPad Prism 7. Dose-response relationships were evaluated through Spearman’s rank correlation. The parallelism of dose-response curves was assessed by the parallel line method: when the slopes of the curves for O-Ac and non-OAc O-Ags obtained by log-transforming ELISA or SBA results vs. log transformed antigen doses were not significantly different from each other, comparison of the Y-intercepts was performed.

## 3. Results

Using the simulation data for SF Y, Sf 6n, Sf 6 and the O-acetylated Sf 6-3Ac and Sf 6-4Ac, we first compare the chain flexibilities of the O-Ags, then consider the molecular conformations, the characteristics of the molecular surface and finally contrast the minimal binding epitopes. In light of these results, we then consider the effect of O-acetylation on Sf 6 immunogenicity in mice.

### 3.1. Chain Extension and Flexibility

**Figure 2:**
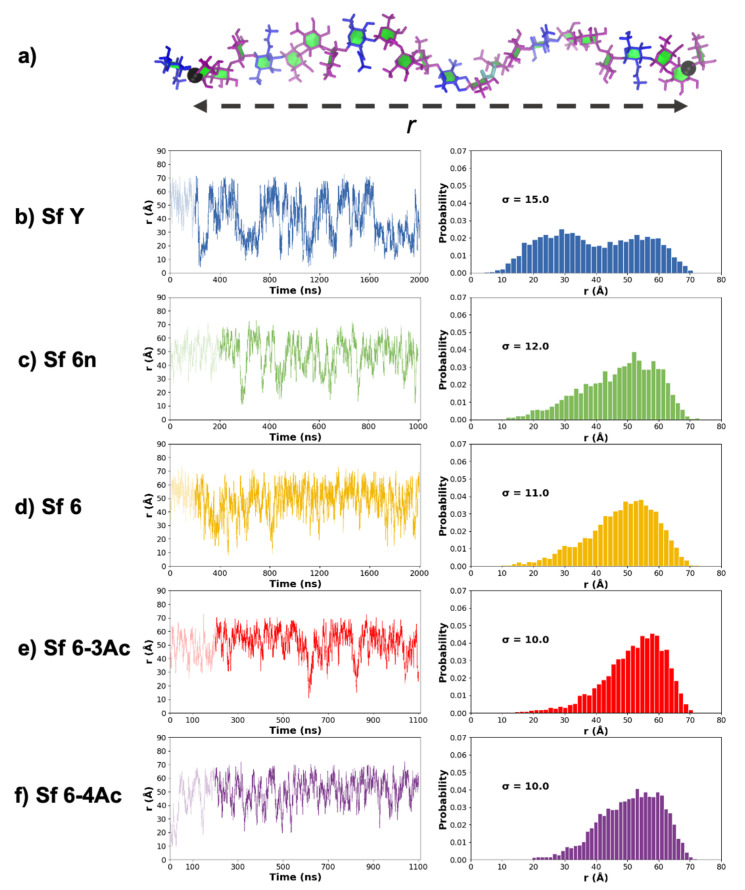
End-to-end distance, *r*, is defined as shown in (**a**) with end-to-end distance time series as well as histograms shown for O-Ags (**b**) Sf Y, (**c**) Sf 6n, (**d**) Sf 6, (**e**) Sf 6-3Ac, and (**f**) Sf 6-4Ac. The standard deviation of each histogram, σ, is shown.

Time series plots of the molecular end-to-end distance, *r*, over the course of the MD simulations provide a simple comparison of the molecular flexibility and chain extension of the O-Ags. We defined *r* (illustrated in Figure 2a) as the distance from C2 of Rha^II^ (residue B) in RU 1 to C1 of Rha^I^ (residue C) in RU 6 (for Sf Y), or the equivalent C1 of GalA in RU 6 (for Sf 6′s). Comparison of the *r* time series plots (Figure 2 left column) and corresponding histograms (Figure 2 right column) reveals significant differences between the Sf Y and Sf 6 O-Ags. Sf Y (Figure 2b) is the most flexible, with the greatest variance (σ = 15) and range (10 Å to 70 Å) of *r*. Further, *r* has a bimodal distribution for Sf Y (with peaks at 25 Å and 52 Å) that is markedly different to the unimodal, left skewed distributions of the Sf 6′s: the Sf Y backbone has a significant population of compact conformations that is absent in the Sf 6′s. In contrast, the distribution of *r* across the four variations of Sf 6 is remarkably similar: the graphs show that the chains are predominantly extended and that the addition of charge or O-acetylation to the Sf 6 backbone has only a minor effect on the O-Ag chain extension and flexibility. The uncharged Sf 6n (Figure 2c) is the most flexible Sf 6 O-Ag with the largest range of *r* (15 Å to 70 Å, σ = 12). Sf 6 (Figure 2d) has a similar *r* distribution (10 Å to 70 Å, σ = 11), while the O-acetylated Sf 6-3Ac (Figure 2e) and Sf 6-4Ac (Figure 2f) are the most extended chains and show some reduction in flexibility (σ = 10). Overall, flexibility of the O-Ags decreases in the order Sf Y >> Sf 6n > Sf 6 > Sf 6-4Ac > Sf 6-3Ac.

### 3.2. Molecular Conformations

**Figure 3:**
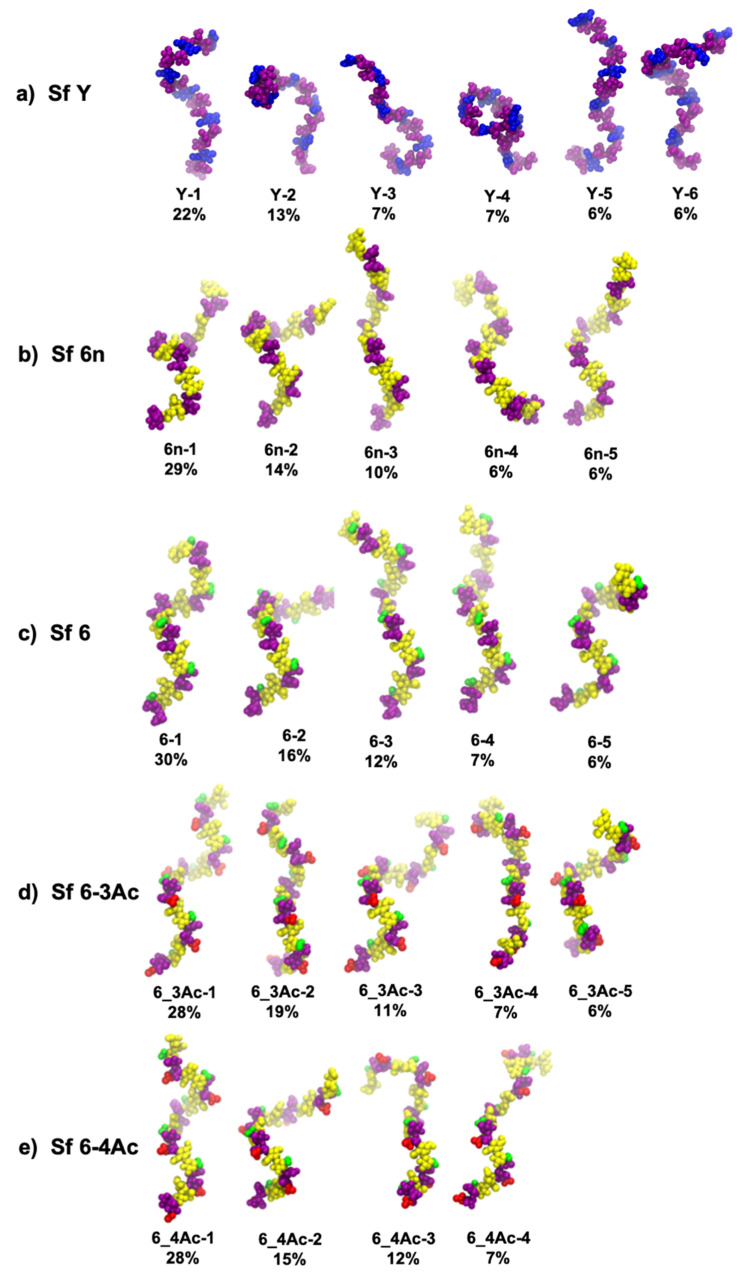
Dominant conformations of the middle 4 repeating units of the 6 RU O-Ag chains for *S. flexneri*: (**a**) Sf Y, (**b**) Sf 6n, (**c**) Sf 6, (**d**) Sf 6-3Ac, and (**e**) Sf 6-4Ac. The relative occupancies of the conformational clusters (excluding the initial 200 ns of equilibration) are shown as percentages with clusters less than 6% not shown. The color scheme is as follows: blue for Glc and GlcNAc; purple for Rha; yellow for Gal, GalA, and GalNAc; green for the COOH group of GalA; red for the O-acetyl groups.

Figure 3 provides a comparison of the dominant chain conformations for Sf Y and the Sf 6 O-Ag, as determined by cluster analysis. While the wide range of conformations demonstrate that all the O-Ags are very flexible and mostly extended, the Sf Y backbone adopts compact conformations (Figure 3a: Y-2, Y-4, Y-6) that do not occur in any of the Sf 6′s (Figure 3b–e). Further, while Sf Y moves between extended and collapsed, helical, and non-helical conformations, the Sf 6 chains adopt the same helical structure in different stages of elongation or compression. Furthermore, the Sf Y helices (Figure 3a: Y-1) are right-handed, whereas the Sf 6′s helices are all left-handed. We investigated the structural basis of the change in handedness of the helix by building 6 repeating unit static structures of Sf Y with our CarbBuilder software, each with one of the structural modifications seen in serotype 6′s backbone (supplementary Figure S2). Of these static structures, only one change, the replacement of the αLRha(1→3)αLRha linkage with the αLRha(1→4)βDGalA linkage (the BC linkage), caused a shift from a right handed helix to left handed, indicating that the change in this linkage position and configuration causes the change in handedness of the helix. This is also apparent in a comparison of heatmaps of the φ and ψ dihedral angles (Figure S3 and Table S1) of Sf Y and Sf 6. The occupancies of the AB and DA linkages are mostly unchanged, whereas the shape and location of the BC linkage is very different in Sf 6 with a small change also seen in the adjacent CD linkage.

Substitutions to the Sf 6 backbone have some small conformational effects, as follows. The addition of charge on Gal slightly elongates the helices: compare the primary conformation of the neutral Sf 6n O-Ag (Figure 3b, 6n-1) with the charged Sf 6 (Figure 3c, 6-1). The greater steric hindrance of the charged GalA monosaccharide versus the neutral Gal is likely the reason for the greater extension observed in the charged Sf 6 O-Ag, also accounting for the neutral Sf 6n being more flexible than the charged Sf 6 as seen above. However, O-acetylation does not have a large conformational impact. The primary conformations of Sf 6-3Ac (Figure 3d) and Sf 6-4Ac (Figure 3e) are very similar extended helices–compare 6_3Ac-1, 6_4Ac-1 with 6-1. Of interest is that all the backbone substitutions-the charged carboxylic acid group on GalA and the O-acetyl groups-are situated on the outside of the helix where they may be easily accessible for antibody binding. In addition, the O3 acetyl group interacts more with the chain backbone than the O4 acetyl, due to its close proximity to the linkage position at C2.

### 3.3. Molecular Surface

While similar in conformation, the O-Ags differ in charges and substituents. Comparison of the hydrophilic and hydrophobic regions of the molecular surface in the O-Ags highlights differences between the O-Ags that may affect antibody binding.

**Figure 4:**
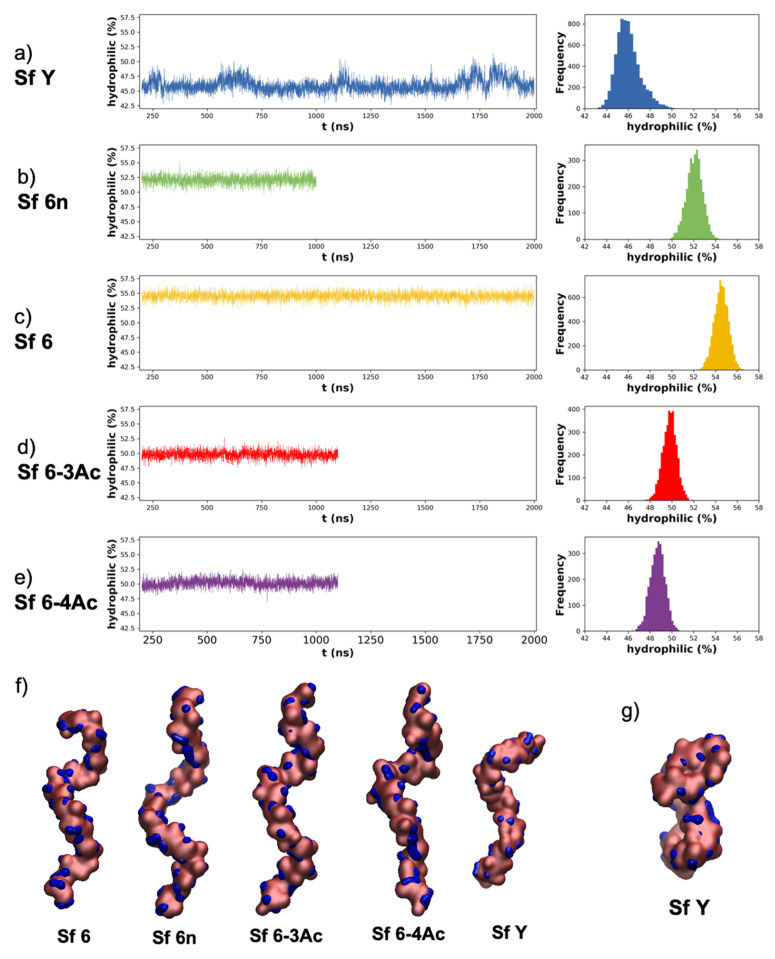
Time series and histogram plots of relative % hydrophilic surface area for O-Ags: (**a**) Sf Y, (**b**) Sf 6n, (**c**) Sf 6, (**d**) Sf 6-3Ac, (**e**) Sf 6-4Ac. VMD *Quicksurf* representation of the hydrophilic (blue) and combined neutral/hydrophobic (pink) surfaces of the O-antigens are shown in (**f**) ordered from highest (Sf 6) to lowest (Sf Y) % hydrophilic surface. (**g**) VMD *Quicksurf* representation of a collapsed Sf Y conformation from around. Note that for Sf Y and Sf 6, longer simulation times were required to reach convergence (both 2000 ns) than for Sf 6n (1000 ns), Sf 6-3Ac (1100 ns), and Sf 6-4Ac (1100 ns).

Time series and histograms of the hydrophilic surface area of each frame of the trajectory are plotted in Figure 4, where hydrophilic surface area refers to the percentage of the surface area that is hydrophilic relative to total solvent accessible surface area. Sf Y (Figure 4a) is markedly more hydrophobic than Sf 6n (Figure 4b) and Sf 6 (Figure 4c), and also shows a greater variation in surface hydrophilicity. This is because the extended conformations of the backbone (Figure 4f) expose more hydrophobic patches (blue) than the collapsed conformations of Sf Y (Figure 4g). The hydrophobicity of Sf Y relative to the Sf 6′s can be rationalized by considering the surfaces of the individual constituent monosaccharides of the backbone (Figure S4)–Gal is markedly more hydrophilic than Glc or Rha. Overall, the hydrophilicity of the exposed O-Ag surface decreases in the order: Sf 6 (55%) > Sf 6n (52%) > Sf 6-3Ac (51%) > Sf 6-4Ac (50%) > Sf Y (46%).

Unsurprisingly, the charged Sf 6 is more hydrophilic than the neutral O-Ag (Sf 6n). It is also clear that O-acetylation decreases the hydrophilicity of the chain: with Sf 6-3Ac (Figure 4d) showing a smaller decrease (4%) than Sf 6-4Ac (5%, Figure 4e) which has the O-methyl more exposed on the chain.

### 3.4. Minimal Binding Epitope

**Figure 5:**
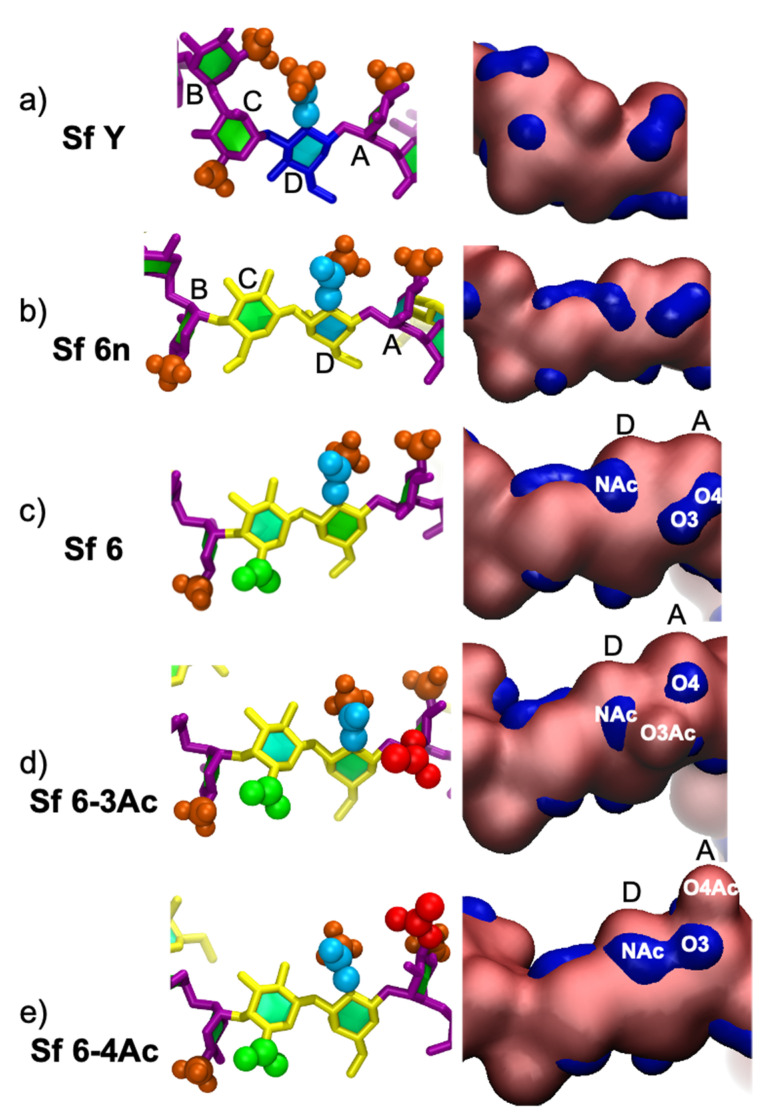
Close up of BCDA residues of RU3/4 in the licorice/paperchain/VDW and *Quicksurf* representations of the primary clusters of: (**a**) Sf Y, (**b**) Sf 6n, (**c**) Sf 6, (**d**) Sf 6-3Ac, and (**e**) Sf 6-4Ac O-antigens. The licorice/paperchain/VDW colors: purple- Rha; dark blue—Glc; yellow—Gal; green—COOH; red—O-acetyl; orange—Me; and cyan—electron withdrawing portion of NAc. The *Quicksurf* colors: blue—hydrophilic surface, pink—hydrophobic & neutral surface.

As antibodies bind to O-Ag regions comprising one to seven residues [27], it is useful to consider the differences in the 6 RU O-Ag’s on this length scale. Figure 5 compares the main conformation and the surface hydrophilicity of a central four residue (BCDA) segment across the Sf Y and Sf 6 O-Ags. It is clear that Sf Y (Figure 5a) has a very different conformation to the SF 6′s. The Sf Y repeting unit is more condensed, residue B is rotated 180°, and the residue B methyl (Me) group interacts with the N-Acetyl on residue D. The surface representations for Sf Y also show smaller regions of hydrophilicity (blue) compared to the Sf 6′s

The Sf 6′s (Figure 5b–d) all have similar conformations of the central BCD fragment; the methyl (Me) on residue B interacts with the hydroxyl (OH) or carboxylic acid (COOH) group on C6 of residue C. The substitutions on the Sf 6 backbone do not affect the orientation of this fragment significantly, but they do affect the DA fragment and the binding surface. Relative to Sf 6 (Figure 5c), O-acetylation at O3 in Sf 6-3Ac (Figure 5d) or O4 in Sf 6-4Ac (Figure 5e) results in a disruption of the hydrophilic regions (blue) of residue A (O3 and O4 region) and residue D (NAc region). For the residue A region, the substituted position (O3 or O4) becomes hydrophobic, reducing the size of the local hydrophilic region and, in the case of Sf 6-4Ac, shifting upwards due to a change in orientation of the O-acetyl to reduce steric hindrance. The residue D region (associated with NAc) shifts towards residue A in Sf 6-3Ac due to NAc–O3Ac interactions and in Sf 6-4Ac, merges with the O3 hydrophilic region.

Overall, the differences between Sf Y and Sf 6 orientations and hydrophilic surfaces indicate the likely antigen binding sites are significantly different. Furthermore, this four-residue region is very similar in the Sf 6′s, suggesting that that O-acetylation does not have a significant effect on the potential antigen binding site, and hence immunogenicity, of the Sf 6 O-Ag.

### 3.5. The Impact of O-Acetylation on the Immunogenicity of Sf 6 GMMA in Mice

To investigate the impact of O-acetylation on the immune response induced by the Sf 6 O-Ags (and verify the predictions made by modeling) the GMMA-producing strain Sf 6 Sh10.8537 Δ*tolR* was further mutated to abolish Rha^III^ O-acetylation by removing the *oacC* gene-coding for the O-acyltransferase C enzyme-responsible for the backbone modification that confers the presence of O-factor 9. Both GMMA we free of soluble proteins and DNA as detected by HPLC-SEC analysis. The O-Ag to protein weight ratio was 0.4 in the wild type GMMA and 0.23 in Δ*oacC* GMMA. The O-Ag chains on both GMMA had three main populations at average sizes of 174 kDa (G4C), 22 kDa and 1.7 kDa [20], respectively. ^1^H NMR analysis of the O-Ag extracted from the Δ*oacC* GMMA confirmed the absence of O-acetylation on Rha^III^, while the O-Ag from the wild type GMMA was O-acetylated at positions 3 and 4 of Rha^III^ (73.3% and 17.9%, respectively), as shown in Figure S5.

The characterization of the resulting GMMA by ^1^H NMR analysis (Figure S5) confirmed the absence of O-acetylation on Rha^III^. Moreover, the % O-acetylation at positions 3 and 4 of Rha^III^ of the native O-Ag were calculated as 73.26% and 17.95%, respectively.

**Figure 6:**
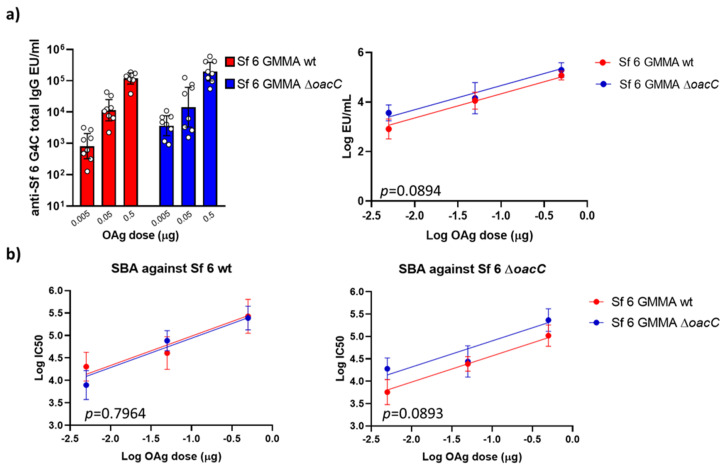
Immunogenicity in mice of *S. flexneri* 6 (Sf) GMMA differing in O-Ag O-acetylation on Rha^III^. Eight CD1 mice per group were immunized intraperitoneally with three doses of O-Ag (0.005, 0.05, 0.5 μg). *S. flexneri* 6 Group 4 Capsule (G4C), encompassing the same RU of the O-Ag, was used as the ELISA coating antigen. (**a**) Summary graph of anti-G4C specific IgG geometric mean units (bars) and individual antibody levels (dots) on the left; statistical comparison of the resulting dose response curves on the right; (**b**) Summary graphs of SBA IC50 titers against *S. flexneri* 6 wild type (on the left) and Δ*oacC* (on the right) strains. ELISA and SBA data were analyzed using the parallel line approach. Each curve represents log-transformed doses on the abscissa and the log-transformed ELISA units or SBA titers on the ordinate. The parallelism of the lines was tested by comparison of the slopes, which resulted in no significant differences. Subsequently, the Y-intercept of the curves were compared, and the *p*-values are reported in the graphs.

The immunogenicity of GMMA displaying O-Ag with or without O-acetylation on Rha^III^ was tested in a dose ranging study in mice, comparing GMMA constructs at the same O-Ag dose. Analysis of sera collected two weeks after the second immunization at day 28 (day 42) showed that wild type and *ΔoacC* GMMA did not induce significantly different anti-O-Ag total IgG response in the dose range tested (Figure 6a).

SBA analysis against both wild type and Δ*oacC S. flexneri* serotype 6 strains confirmed the results obtained in ELISA, indicating additionally, that the functionality of the antibodies induced was not affected by O-Ag O-acetylation (Figure 6b).

## 4. Discussion

Our simulations show that the Sf Y and Sf 6 O-Ags are very flexible, in common with serogroups 2, 3, and 5 modeled previously. However, while Sf Y adopts both elongated and collapsed structures, including right-handed helices, the Sf 6 O-Ags are predominantly in an extended left-handed helical conformation. This conformational change is a result of the change of the BC linkage from a flexible equatorial configuration in Sf Y to a more constrained axial configuration in Sf 6. Due the significant conformational differences between the Sf Y backbone and Sf 6, cross-protection between Sf 6 and Sf Y is not expected to occur.

O-acetylation is known to alter carbohydrate chemical and physical properties, such as molecular conformation and hydrophobicity, thereby affecting the antigenicity and immunogenicity of antigens of relevance for vaccine design [51]. Recent reviews on *Shigella* vaccine development [26] and the role of O-acetylation [51] confirm its contribution to the functional immune response for some licensed bacterial polysaccharide-based vaccines (*Salmonella typhi* Vi and *Neisseria meningitidis* serogroup A), but not for others, indicating that the importance of O-acetylation must be established on a case-to-case basis. For *Shigella*, O-acetylation is recognized as a source of additional antigenic diversity and has been characterized by group O-factors [23]. In the case of Sf 6, the presence of Rha^III^3/4Ac introduces group O-factor 9 and three subtypes have been recognized on the basis of levels of O-acetylation (I and II) and its absence (III) [51]. Further, the presence of an O-acetylated rhamnobiose (αLRhaIII3/4Ac(1→3)αLRha^II^) has been suggested as the structural basis for the observed cross-reactivity between Sf 2a and SF6 [18,51].

Our simulations show that O-acetylation of Sf 6 at O3 or O4 of Rha^III^ (residue A) does not alter the backbone conformation significantly, suggesting that O-acetylation may not be essential for antigenicity. However, O-acetylation results in a significant decrease in the hydrophilicity of the O-Ag in Sf 6 compared to de-O-acetylated Sf 6 based on solvent accessible surface area calculations. This may affect the binding affinity of Sf 6 compared to de-O-acetylated Sf 6, however, the biological significance of this parameter is yet to be established.

Indeed, our comparison of Sf 6 O-Ags with and without O-acetylation on Rha^III^ (delivered on GMMA) in mice confirmed that O-acetylation does not play a major role in the ability of the O-Ag to induce antibodies able to recognize the O-acetylated RU and to kill O-acetylated or de-O-acetylated *S. flexneri* 6 bacteria. The results obtained in vivo corroborate the modeling analysis, showing no impact of O-acetylation on immunogenicity. This is in line with the marginal difference observed in the O-Ag conformation. These results are also in agreement with our studies on CRM_197_ glycoconjugates [20].

Further validation of the importance of the O-acetylation and cross reactivity of *S. flexneri* serotype 6 in other animal models (and preferably humans) is necessary to definitively confirm the role of O-acetylation in immunogenicity and provide insight into appropriate, representative animal models for *Shigella* sp vaccine development.

## Data Availability

Not Applicable.

## Supplementary Materials

The following are available online at https://www.mdpi.com/article/10.3390/vaccines9050432/s1, Figure S1: Blocked standard error calculations for determining the extent of simulation convergence, Figure S2: Static structures for establishing the origin of the handedness of the *S. flexneri* serotype 6 backbone, Figure S3: Heat map representation of scatter plots for the phi (φ), psi (ψ) dihedral angles of glycosidic linkages of *S. flexneri* serotype Y and 6 backbones, Table S1: Tabulation of primary and secondary occupied dihedral angles of glycosidic linkages for the modelled O-antigens, Figure S4: Hydrophilic/hydrophobic surface visualization of *S. flexneri* serotype Y and 6 constituent monosaccharides, Figure S5: Diagnostic ^1^H NMR spectra of *S. flexneri* 6 O-Ags extracted from GMMA produced by the wild type and Δ*oacC* mutant strains.
