# Supplementary material for: Conformational and Immunogenicity Studies of the Shigella flexneri Serogroup 6 O-Antigen: The Effect of O-Acetylation
Source: Vaccines (Basel). 2021 Apr 27;9(5):432. doi: 10.3390/vaccines9050432 (PMC8144980; doi:10.3390/vaccines9050432)
Supplement: Supplementary file 1 [file vaccines-09-00432-s001.zip › vaccines-1190132-supplementary.pdf]

vaccines

Supplementary

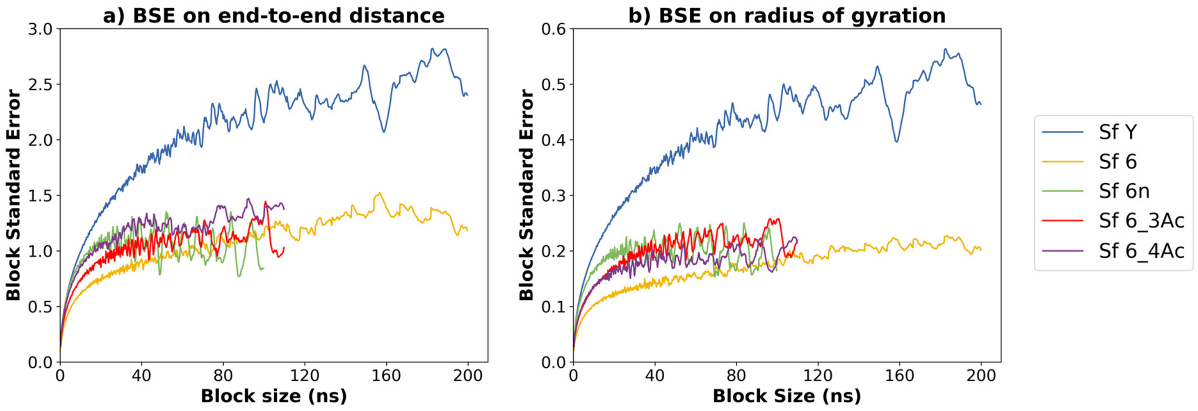

Figure S1. Block standard averaging analysis calculated for the time series of a) end-to-end distance and b) radius of gyration of the 6RU O-antigen chains. The blocked standard error (BSE) reaches a plateau with increased block size (ns) for all of the O-antigens indicating convergence. Further analysis using the approximate BSE reveals correlation times < 55 ns (much less than the simulations times of 1000-2000 ns) and numbers of independent samples >> 1 which support the convergence of the simulation.

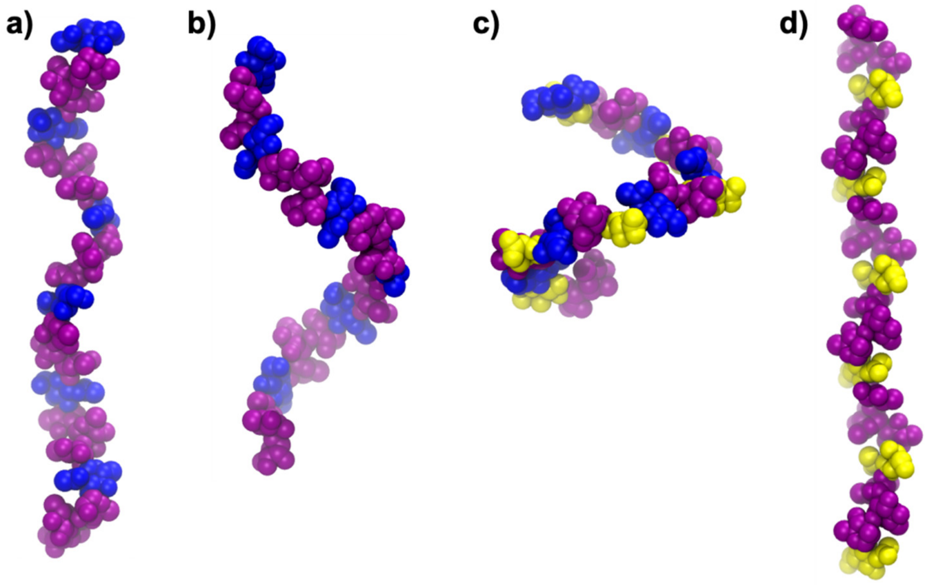

Figure S2. 6 repeating unit static structures of the Sf Y repeating unit, each with one of the structural modifications seen in serotype 6's backbone: a) Sf Y backbone with no replacement, b) replacing the BC (αLRha1→3αLRha) linkage with an (αLRha1→4αLRha) linkage, c) replacing residue C, rhamnose, with galactose (linkages unchanged), d) replacing residue D, GlcNAc, with GalNAc (linkages unchanged). Only structure b) changes from a right-handed to left-handed helix indicating the change in linkage position (which involves changing from an equatorial to axial linkage) causes the change in handedness of the helix.

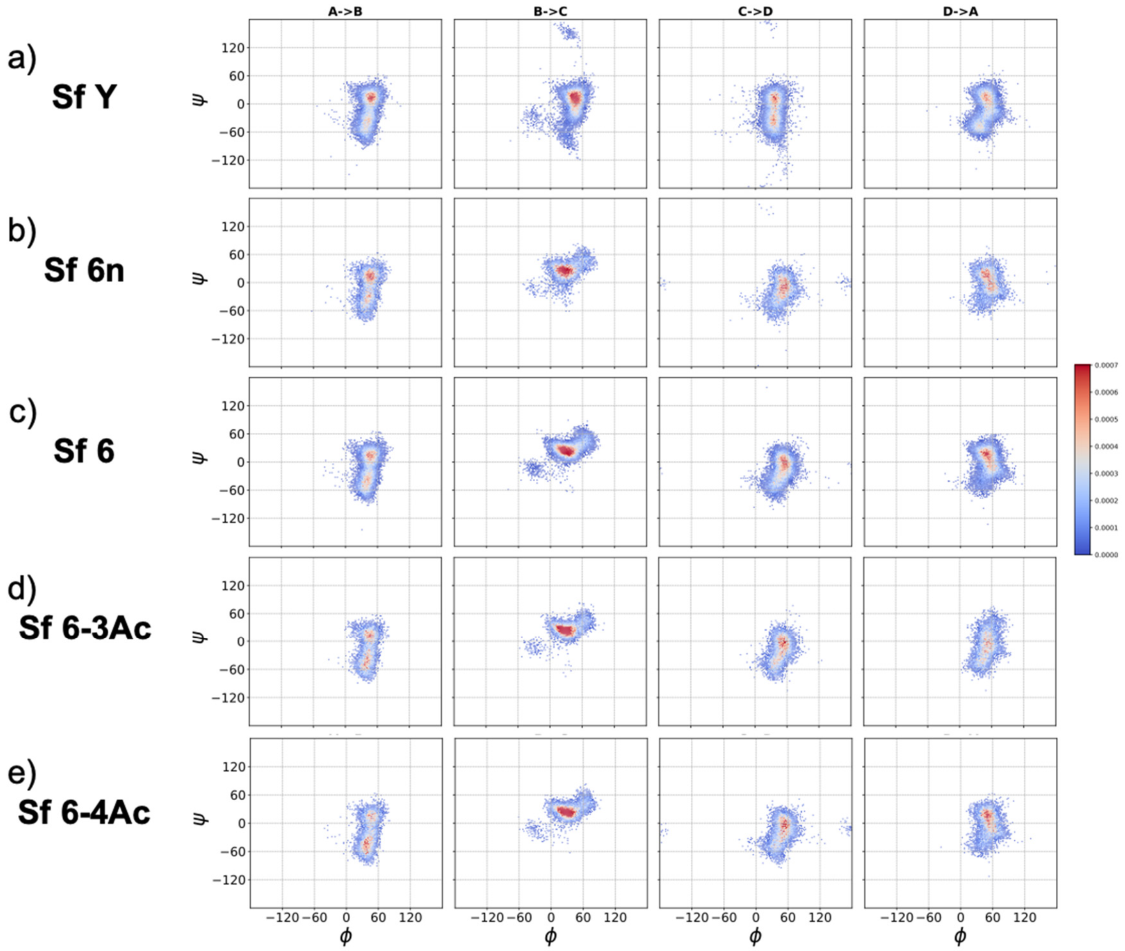

Figure S3. Heat map representation of scatter plots for the phi (ϕ), psi (ψ) dihedral angles of each glycosidic linkage for: (**a**) Sf Y, (**b**) Sf 6n, (**c**) Sf 6, (**d**) Sf 6-3Ac, (**e**) Sf 6-4Ac. Changes in chain conformation are primarily caused by changes in the orientations of the constituent glycosidic linkages due to the carbohydrate rings being quite constrained. Points from both of the central repeating units (RU 3 and RU 4) were included to broadly sample the backbone behaviour. The color scale indicates the relevant occupancy of the dihedral angles during the simulation with red being high occupancy, and blue low occupancy.

Table S1. Tabulation of primary and secondary occupied dihedral angles of glycosidic linkages for the modeled O-antigens.

O-antigen	Glycosidic Linkage (of repeating unit ABCD)						
	AB		BC	CD		DA	
	Primary (φ, ψ)	Secondary (φ, ψ)	Primary (φ, ψ)	Primary (φ, ψ)	Secondary (φ, ψ)	Primary (φ, ψ)	Secondary (φ, ψ)
**Sf Y**	50, 15	40, -45	45, 10	40, 10	40, -45	50, 10	40, -45
**Sf 6n**	45, 15	40, -30	25, 25	55, -10	N/A	50, 15	60, -10
**Sf 6**	45, 15	40, -45	30, 20	55, 0	N/A	50, 15	N/A
**Sf 6-3Ac**	45, 15	40, -45	25, 25	55, -5	N/A	55, -5	N/A
**Sf 6-4Ac**	45, 15	35, -45	25, 25	55, -5	N/A	55, 15	N/A

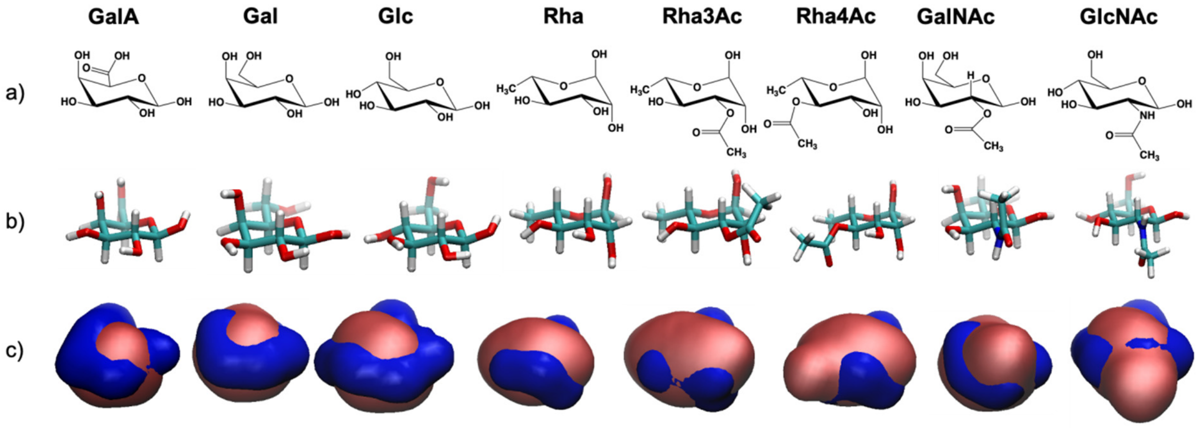

Figure S4. (**a**) line structures of *S. flexneri* RU constituent monosaccharides, (**b**) CPK models of the monosaccharides, (**c**) Quicksurf representation of monosaccharides with hydrophilic regions in blue and hydrophobic/neutral regions in pink. Monosaccharides are arranged in order of decreasing % hydrophilic surface: GalA, 74%; Gal, 68%; Glc, 62%; Rha, 60.4%; Rha3Ac, 60.2%; Rha4Ac, 59.3%; GalNAc, 59%; GlcNAc, 58%.

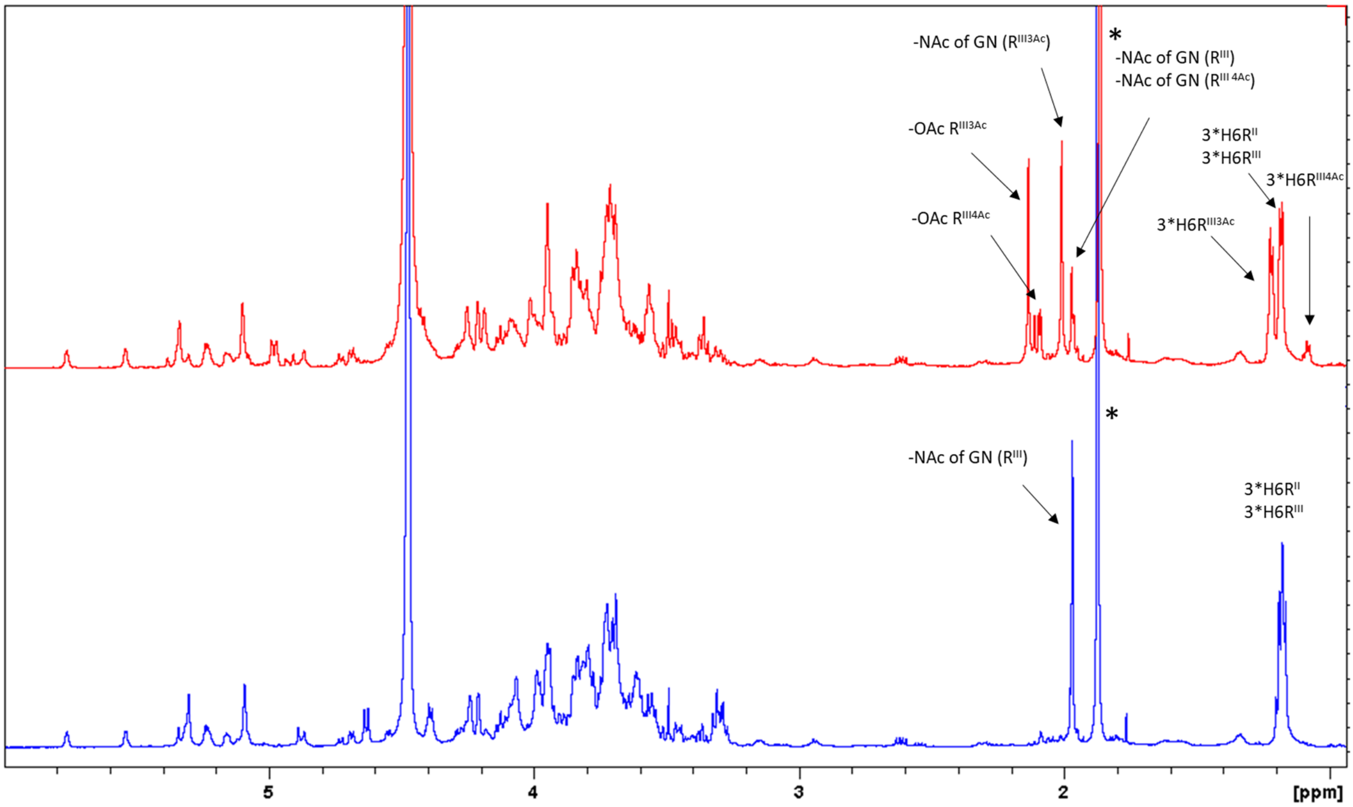

Figure S5. ^{1}H NMR spectra (from 1 to 6 ppm) of *S. flexneri* 6 O-Ags extracted from GMMA produced by the wild type (red) and *ΔoacC* mutant (blue) strains, confirming expected O-acetylation patterns. Diagnostic signals are assigned in the spectra.*=acetate buffer. For the annotations, GN = GlcNAc, R = Rha.
